# Single nucleotide polymorphism rs3124599 in Notch1 is associated with the risk of lung cancer in northeast Chinese non-smoking females

**DOI:** 10.18632/oncotarget.16101

**Published:** 2017-03-10

**Authors:** Xiaowei Quan, Zhihua Yin, Xue Fang, Baosen Zhou

**Affiliations:** ^1^ Department of Epidemiology, School of Public Health, China Medical University, Shenyang, China; ^2^ Key Laboratory of Cancer Etiology and Prevention, China Medical University, Liaoning Provincial Department of Education, Liaoning, China

**Keywords:** Notch1, single nucleotide polymorphisms, lung cancer, small cell lung cancer, non-smoking females

## Abstract

Lung cancer is one of the most common cancers and the main cause of cancer-related deaths. Notch1 might play a part in tumorigenesis of lung cancer. Here we explored the relationship of three SNPs (rs3124599, rs3124607 and rs3124594) in Notch1 with the risk and the survival of lung cancer in non-smoking females, including 556 cases and 395 controls. Chi-square tests, logistic regression analysis and crossover analysis were conducted to estimate the association between SNPs and the risk of lung cancer and the interaction between SNPs and environmental exposure. Survival analysis was conducted to explore the association between SNPs and survival of lung cancer. The results demonstrated that the polymorphism of rs3124599 was associated with the susceptibility of lung cancer in recessive model (AA+AG vs. GG). Compared to the those with AA or AG genotype, individuals with GG genotype had a 1.562-fold increased risk of lung cancer (*P* = 0.023, OR = 1.562, 95% CI = 1.062-2.297). In stratified analysis, the GG genotype of rs3124599 would increase the risk of small cell lung cancer (SCLC) (*P* = 0.011, OR = 2.167, 95% CI = 1.193-3.396). However, no significant interaction between rs3124599 and cooking oil fume exposure was observed either in addictive model or multiplicative model. The results of survival analysis showed there was no significant association between SNPs and prognosis of lung cancer (*P* = 0.949 for rs3124599, *P* = 0.508 for rs3124607, *P* = 0.884 for rs3124594). Our study might indicate that rs312599 in Notch1 may be a novel biomarker for SCLC risk in Chinese non-smoking females

## INTRODUCTION

Lung cancer is the most frequently diagnosed cancer and the most common cause of cancer-related deaths in the worldwide. Approximately 1.8 million new lung cancer cases occurred in 2012, which made up about 13% of total cancer diagnoses [1]. Smoking is the major risk of lung cancer [2]. However, in spite of a lower prevalence of using tobacco in China, the rate of lung cancer in Chinese females is higher than in females in some European countries [1], which means other factors, such as genetic risk factors, may play a role in the development of female lung cancer. Meanwhile, since women cook more than men in most Chinese families, exposure of cooking oil fume may increase the risk of non-smoking female lung cancer. This paper studied the association between single nucleotide polymorphisms (SNPs), environmental exposure and non-smoking female lung cancer risk and survival.

Notch1 is located in 9q34.3 and encodes a member of the NOTCH family of proteins. The dysregulation of Notch1 is associated with the risk, the development, and the prognosis of cancers [3–5]. However, the function of Notch1 is an oncogene or a tumor suppressor depending on the type of tumor and cellular context [6–9]. In non-small cell lung cancer (NSCLC), Notch1 is aberrantly activated, and the activation of Notch1 is associated with progression and poor prognosis of NSCLC [10, 11]. However, in small cell lung cancer (SCLC), overexpression of Notch1 can cause G1 cell cycle block and can inhibit epithelial-to-mesenchymal transition (EMT), cell motility and cell metastatic potential [12, 13]. Extensive studies have suggested that the alteration of Notch1 expression is involved in the development of diseases. However, what are more important in pro-oncogenic role are mutations in Notch1. More than 50% of human T cell acute lymphoblastic leukemia (T-ALLs) have active mutations in Notch1 [14]. Mutations in Notch1 are also associated with many other diseases, such as aortic valve disease [15], head and neck squamous cell carcinoma [16], and breast cancer [17]. Nevertheless, few studies focus on the association between mutations in Notch1 and lung cancer, especially non-smoking female lung cancer.

In the present study, the association between some SNPs in Notch1 and the susceptibility and survival of lung cancer was evaluated, and the interaction between SNPs in Notch1 and environmental factors related with the risk of lung cancer was investigated to provide a new lung cancer screening strategy in non-smoking females.

## RESULTS

### Subjects characteristics

The demographic clinical characteristics of 556 cases and 395 controls were listed in Table 1. There were no significant differences in the distribution of the age (*P* = 0.426) between cases and controls with a similar means of age (56.74 ± 11.70 years for cases and 56.13 ± 11.64 years for controls). Cases included 96 squamous cell carcinomas, 371 adenocarcinomas and 89 small cell carcinomas.

**Table 1:** Demographic and clinical characteristics of subjects in Northeast Chinese non-smoking female population

Variables	Case	Control	*P* value
Age			0.911
< 60	302	216	
≥ 60	254	179	
Stage			
I	22		
II	136		
III/IV	398		
Pathological type			
Adenocarcinoma	371		
Squamous carcinoma	96		
Small cell lung cancer	89		
Family history of cancer*			0.675
Yes	29	11	
No	264	117	
Cooking oil fume exposure*			0.112
Yes	91	30	
No	202	98	

*There were missing values.

### SNP frequencies and association with lung cancer

SNP frequencies and their association with lung cancer were listed in Table 2 and Supplementary Table 1. The genotype frequencies of rs3124599 (χ^2^ = 2.37, *P* = 0.12), rs3124607 (χ^2^ = 3.65, *P* = 0.06), rs3124594 (χ^2^ = 0.06, *P* = 0.81) in controls were conformed to Hardy-Weinberg equilibrium. For the distribution of rs3124599, significant difference was observed between AA genotype and GG genotype (Table 2). Homozygous carriers of GG genotype had a 1.595-fold elevated risk of lung cancer than homozygous carriers of AA genotype. According to the results of further analysis, the distribution of rs3124599 was prominently different between cases and controls in recessive model. Individuals with GG genotype, including 57 patients with adenocarcinoma, 15 ones with squamous carcinoma and 19 ones with SCLC, had a 1.562-fold increased risk of lung cancer than those carrying AG or AA genotype. However, no significant association was observed between rs3124607, rs3124594 and the risk of lung cancer in genotype comparisons or allele comparisons (Supplementary Table 1).

**Table 2:** Distribution of rs3124599 and lung cancer risk

SNP	Case (%)	Control (%)	OR (95% CI)*	*P* value
rs3124599				
AA	201 (36.2%)	155 (39.2%)	1.000	
AG	264 (47.5%)	196 (49.6%)	1.038 (0.785,1.373)	0.794
GG	91 (16.4%)	44 (11.1%)	1.595 (1.052,2.419)	0.028
Dominant model				
AA	201 (36.2%)	155 (39.2%)	1.000	0.334
AG+GG	355 (63.8%)	240 (60.8%)	1.140 (0.874,1.487)	
Recessive model				
AA+AG	465 (83.6%)	351 (88.9%)	1.000	0.023
GG	91 (16.4%)	44 (11.1%)	1.562 (1.062,2.297)	
Allele model				
A			1.000	0.066
G			1.193 (0.988,1.441)	

*OR was adjusted by age.

To further investigate the association between rs3124599 and risk of lung cancer, stratification analysis was conducted in recessive model. The AA genotype and the AG genotype, without mutant allele, were considered as a reference. The results in Table 3 suggested that rs3124599 might have an effect on the risk of SCLC and individuals with GG genotype had a 2.167-fold increased risk of SCLC. Among patients who were younger than 60, carriers of GG genotype had a 1.583-fold elevated risk of lung cancer, while no significant difference was observed among patients who were older than 60.

**Table 3:** Stratification analysis of rs3124599 polymorphisms and risk of lung cancer in recessive model

Variables	Case (%)	Control (%)	OR (95% CI)	*P* value
Pathological type				
Adenocarcinoma				
AA+AG	314 (84.6%)	351 (88.9%)	1.000	—
GG	57 (15.4%)	44 (11.1%)	1.452 (0.952–2.214)*	0.083
Squamous carcinoma				
AA+AG	81 (84.4%)	351 (88.9%)	1.000	—
GG	15 (15.6%)	44 (11.1%)	1.489 (0.788–2.815)*	0.220
Small cell lung cancer				
AA+AG	70 (78.7%)	351 (88.9%)	1.000	—
GG	19 (21.3%)	44 (11.1%)	2.167 (1.193–3.396)*	0.011
Age				
< 60				
AA+AG	252 (83.4%)	351 (88.9%)	1.000	—
GG	50 (16.6%)	44 (11.1%)	1.583 (1.023,2.448)	0.039
≥ 60				
AA+AG	213(83.9%)	351 (88.9%)	1.000	—
GG	41 (16.1%)	44 (11.1%)	1.536 (0.971,2.428)	0.067

*OR was adjusted by age.

### Interaction between rs3124599 and cooking oil fume exposure

In consideration of the fact that genetic factors could interact with environmental exposures in some cases, we investigated the interaction between rs3124599 and cooking oil fume exposure by crossover analysis and logistic regression model. The results of crossover analysis demonstrated that there was no significant interaction between rs3124599 and cooking oil fume exposure (Tables 4, 5). The logistic regression model also suggested no interaction exited between rs3124599 and cooking oil fume exposure (*P* = 0.928, OR = 1.072, 95%CI = 0.239–4.812) (Table 6).

**Table 4:** Interaction between rs3124599 and environmental exposures in northeast Chinese non-smoking female population

rs3124599	Cooking oil fume exposure	Case	Control	OR95%CI*	*P* value
AA+AG	—	167	87	1.000	
AA+AG	+	76	27	1.146 (0.875,2.436)	0.147
GG	—	35	11	1.672 (0.808,3.460)	0.166
GG	+	15	3	2.568 (0.721,9.151)	0.146

*OR was adjusted by age.

**Table 5:** Crossover analysis of interaction between rs3124599 and environmental exposures

Measure	Estimate	Lower	Upper
RERI	0.052	–2.138	2.243
AP	0.051	–2.029	2.132
S	–0.509	—	—

**Table 6:** Logistic model of interaction between rs3124599 and environmental exposures

Variables	OR95%CI*	*P* value
rs3124599	1.658(0.803,3.424)	0.172
cooking oil fume	1.466(0.881,2.442)	0.141
interaction	1.072(0.239,4.812)	0.928

*OR was adjusted by age.

### Survival analysis

The relationship between SNPs and survival of lung cancer was summarized in Table 7. No significant association was observed between SNPs and survival of lung cancer. There was no significant difference in the distribution of SNPs in different stages (Supplementary Table 2).

**Table 7:** Association between SNPs and survival of lung cancer in Chinese non-smoking females

SNPs	Case	Death	MST (months)	log-rank *P* value	HR(95%CI)
rs3124599					
AA	120	100	25.510	0.949	1.000
AG	167	143	24.570		0.966 (0.687–1.358)
GG	57	50	24.985		1.006 (0.729–1.388)
rs3124607					
AA	265	230	24.562	0.508	1.000
AG	74	60	26.014		1.244 (0.837–1.850)
GG	5	3	32.400		1.137 (0.751–1.723)
rs3124594					
GG	275	230	25.241	0.884	1.000
AG	66	60	24.046		1.006 (0.470–2.155)
AA	3	3	25.333		1.033 (0.681–1.565)

## DISCUSSION

Genetic mutations and SNPs play a crucial role in tumorigenesis, cancer development, and prognosis of tumor. SNPs could change the sequence of gene products, regulate the expression of genes, or affect the function of genes to alter the phenotype. In the current study, we estimated the relationship between three SNPs in Notch1 and the risk of lung cancer among 951 non-smoking females, including 556 cases and 395 controls. The results suggested that polymorphism of rs3124599 in Notch1 was associated with the risk of lung cancer in non-smoking females, especially with the risk of SCLC. The individuals who carry GG genotype have more risk of SCLC than those who carry AA genotype.

rs3124599, rs3124607 and rs3124594 are located in intron region of Notch1 gene and they cannot change the sequence of the protein, but the three SNPs might regulate the transcription of Notch1 or other genes. rs3124594 is a cis-eQTL (expression quantitative trait locus) of DNLZ gene and INPP5E gene [18, 19]. Polymorphism of rs3124594 could influence the expression of DNLZ and INPP5E, and then influence the mitochondrial HSPA9 catalytic activity [20] and multiple aspects of cellular metabolism. According to the prediction of RegulomeDB and F-SNP database, rs3124607 and rs3124599 might affect transcription factor binding [21, 22]. So the three SNPs could have biological impact on Notch1 signaling or other genes, which will be investigated in our future study.

The minor allele frequency of rs3124599 in controls is 0.359, which is consistent with the data in Hapmap (0.383 for Chinese population). In accordance with the previous studies, SNPs in Notch1 occurred in multiple types of tumor, which could serve as predictive biomarkers for the susceptibility and survival [23, 24]. Liu et al. found that rs13999482 in Notch1 was relevant to the occurrence and development of head and neck squamous cell carcinoma [16]. The study of Cao demonstrated that rs3124591 TC genotype was correlated with high expression of Notch1 which was associated with the development of invasive ductal breast cancer [17].

In stratification analysis of different pathological types, the association between polymorphisms of rs3124599 and the risk of SCLC was observed. GG genotype carriers had a 2.193-fold risk of SCLC with those carrying AA or AG genotype. Previous study demonstrated that Notch1 played a crucial part in carcinogenesis of SCLC. In SCLC, Notch1 serves as a tumor suppressor gene by inhibiting cell growth and regulates cell adhesive and EMT [25]. Overexpression of Notch1 inhibited the processes of EMT and cell metastasis [12]. In addition, knocking down Notch1 gene resulted in the loss of cell adhesion-mediated drug resistance and the SBC-3 SCLC cells lost chemo-resistant ability [26].

Genetic predisposition and environmental factors influence and interact with each other in complex diseases. Thus, interaction between polymorphisms of rs3124599 and cooking oil fume exposure was investigated by logistic model and crossover analysis. The results showed that no significant interaction was observed between rs3124599 and cooking oil fume exposure. One of the reasons might be the information of cooking oil fume exposure is not large enough to obtain a significant difference in statistics.

To the best of our knowledge, the present study is the first one demonstrating that the polymorphism of rs3124599 in Notch1 is associated with the susceptibility of SCLC in northeast Chinese non-smoking female population. However, some limitations should be taken into consideration to draw a conclusion: one is that the size of samples is relatively small, especially in stratification analysis; the other is that the population selected into the present study is from the northeast of China. So a larger and more diversified population is required to verify the results in the future.

In conclusion, our study focused on the relationship between polymorphisms in Notch1 and the susceptibility and survival of lung cancer in non-smoking females in the northeast of China. The present study demonstrated that rs3124599 in Notch1 may be associated with the risk of SCLC in northeast Chinese non-smoking females. However, the conclusion should be verified in a larger population and the biological function of rs3124599 in SCLC should be investigated in the future.

## MATERIALS AND METHODS

### Study subjects

There were 556 cases and 395 controls in the hospital-based case-control section of this study. All the cases were diagnosed newly with histologically confirmed lung cancer between January 2010 and January 2014. The controls who were free from a history of cancer were randomly selected in the same hospital during the same period. Definitions of environmental exposure were in accordance with our previous study [27]. Individuals who smoke less than 100 cigarettes in their lifetime were defined as non-smokers. Participants who engaged in frying or deep-frying in the kitchen more than two times each week with reported eye or throat irritation were defined with cooking oil exposure. All subjects were unrelated ethic Han-Chinese non-smoking females who had signed informed consent forms.

The cases in case-control study were followed up as our previous study reported [28]. Finally, 344 patients’ complete information of death was obtained. This study was approved by the institutional review board of China Medical University.

### DNA isolation and genotyping

A 5ml venous blood sample was obtained from each subject and genomic DNA was extracted by phenol-chloroform method. Genotyping was performed by a 7500 Fast Real-time PCR system (Applied Biosystems, Foster City, CA, USA). PCR Taqman primers and probes were designed by Applied Biosystems (CA, USA) (assay ID C___238631_10). The protocol of the program was heating to 95°Cfor 10min following by 47 cycles of 92°C for 30s and 60°C for 1min. For quality control, the investigators were blinded to the case-control statue and 10% samples were randomly selected to duplicate analysis. The results of the duplicated analysis were totally concordant with the former ones.

Ancestral Allele of rs3124599, rs3124607 and rs3124594 is A, A and G, respectively. So, wild homozygous of rs3124599, rs3124607 and rs3124594 is AA, AA and GG, respectively. Mutant homozygous of these three SNPs is GG, GG and AA, respectively. Heterozygote is AG, AG and AG, respectively.

### Statistical analysis

All statistical analyses were two-tailed and conducted with SPSS software (vision 13.0). A value of *P* < 0.05 was defined as the criterion of statistical significance. Hardy-Weinberg equilibrium (HWE) was tested by a goodness-of-fit χ^2^ test. Student's *t-test* was performed in continued variables and the Pearson's χ^2^ was performed in categorical variables between cases and controls. The odds ratios (OR) and 95% confident intervals (95% CI) were calculated to estimate the association between SNP and the risk of lung cancer by logistic regression analysis after adjustment of age. The interaction between SNPs and environmental exposures was evaluated in multiplicative model and in addictive model. The multiplicative interaction was estimated with OR in logistic regression model. The addictive interaction was estimated with RERI (Relative Excess Risk due to Interaction), AP (Attributable Proportion due to Interaction), and S (Synergy Index). If there was statistically significant interaction, the 95%CI of RERI and AP should not include 0 and the 95%CI of S should not include 1. Survival analysis was performed by log-rank test. HR (Hazard Ratio) and 95%CI were calculated by COX regression analysis.

## SUPPLEMENTARY MATERIALS TABLES


