# Supplementary material for: Single nucleotide polymorphism rs3124599 in Notch1 is associated with the risk of lung cancer in northeast Chinese non-smoking females
Source: Oncotarget. 2017 Mar 10;8(19):31180–6. doi: 10.18632/oncotarget.16101 (PMC5458199; doi:10.18632/oncotarget.16101)
Supplement: Supplementary file 1 [file oncotarget-08-31180-s001.pdf]

Single nucleotide polymorphism rs3124599 in Notch1 is associated with the risk of lung cancer in northeast Chinese non-smoking females

Supplementary Materials

Supplementary Table 1: Distribution of rs3124607, rs3124594 and lung cancer risk

SNPs	Case (%)	Control (%)	OR (95%CI)*	*P* value
rs3124607				
AA	429(77.2%)	310(78.5%)	1.000	
AG	118(21.2%)	84(21.3%)	1.016(0.741,1.394)	0.920
GG	9(1.6%)	1(0.3%)	6.585(0.829,52.284)	0.075
Dominant model				
AA	429	310	1.000	0.622
AG+GG	127	85	1.081(0.792,1.476)	
Recessive model				
AA+AG	547	395	1.000	0.075
GG	9	1	6.562(0.827,52.045)	
Allele model				
A				0.368
G			0.877(0.658,1.168)	
rs3124594				
GG	449(80.8%)	315(79.7%)	1.000	
AG	99(17.8%)	76(19.2%)	0.915(0.657,1.276)	0.602
AA	8(1.4%)	4(1.0%)	1.374(0.410,4.609)	0.607
Dominant model				
GG	449	315	1.000	0.701
AG+AA	107	80	0.939(0.679,1.297)	
Recessive model				
GG+AG	548	391	1.000	0.588
AA	8	4	1.397(0.417,4.678)	
Allele model				
G			1.000	0.838
A			0.969(0.720,1.305)	

*OR was adjusted by age.

Supplementary Table 2: The association between SNPs and clinical stage of subjects

SNPs	Stage			χ2	*P* value
	I	II	III/IV		
rs3124599					
AA	7	51	143	2.132	0.711
AG	11	68	185		
GG	4	17	70		
rs3124607					
AA	19	108	302	4.485	0.344
AG	3	24	91		
GG	0	4	5		
rs3124594					
GG	17	112	320	1.838	0.766
AG	5	21	73		
GG	0	3	5		
